# Bacterial inactivation/sterilization by argon plasma treatment on contaminated titanium implant surfaces:*In vitro* study

**DOI:** 10.4317/medoral.20845

**Published:** 2015-11-22

**Authors:** Marco Annunziata, Luigi Canullo, Giovanna Donnarumma, Pina Caputo, Livia Nastri, Luigi Guida

**Affiliations:** 1 Multidisciplinary Department of Medical-Surgical and Dental Specialties, Second University of Naples, Naples, Italy; 2 Istituto Stomatologico Toscano, Viareggio, Italy; 3 Private Practice in Rome, Rome, Italy; 4 Department of Experimental Medicine, Microbiology Section, Second University of Naples, Naples, Italy

## Abstract

**Background:**

Surface treatment by argon plasma is widely used as the last step of the manufacturing process of titanium implant fixtures before their sterilization by gamma rays. The possibility of using such a technology in the daily clinical practice is particularly fascinating. The aim of the present study was to assess the effects of the argon plasma treatment on different titanium implant surfaces previously exposed *In vitro* to bacterial contamination.

**Material and Methods:**

Sterile c.p. titanium implant discs with turned (T, Sa: 0.8 µm ), sandblasted/acid-etched (SAE, Sa: 1.3 µm) and titanium plasma sprayed (TPS, Sa: 3.0µm) surface were used in this study. A strain of Aggregatibacter *actinomycetemcomitans* ATCC3718 was grown at 37°C under anaerobic conditions for 24 h and then transferred on six discs for each of the three surface types. After 24 hours, a half of the contaminated discs (control group) were directly used to evaluate the colony forming units (CFUs). The other half of the contaminated discs (test group) were treated in an argon plasma chamber for 12 minutes at room temperature prior to be analyzed for CFU counting. All assays were performed using triplicate samples of each material in 3 different experiments.

**Results:**

When the CFU counting was carried out on control discs, a total of 1.50x106±1.4x105, 1.55x106±7.07x104 and 3.15x106±2.12x105 CFU was respectively assessed for T, SAE and TPS discs, without statistically significant differences among the three surfaces. On the contrary, any trace of bacterial contamination was assessed for titanium discs treated in the argon plasma chamber prior to be analyzed, irrespectively to the implant surface tested.

**Conclusions:**

Within the limit of this study, reported data suggested that the argon plasma technology could be efficiently used to decontaminate/sterilize previously infected titanium implant surfaces.

**Key words:**Argon plasma, titanium implant surface, Aggregatibacter actinomycetemcomitans.

## Introduction

Surface treatment by argon plasma is widely used as the last step of the manufacturing process of titanium implant fixtures before their sterilization by gamma rays. This technology works through the activation of the electronic mantle of materials by a spray of argon under pressure at room temperature. The main microscopic effect of such activation is the removal of the microbiologic pollution and contamination from the metallic surfaces. At the same time, this process is also able to modify the physicochemical, and in turn the biological features of implant surfaces, and their interaction with the environment (1-4).

The possibility of using such a technology outside the industrial field in the daily clinical practice is particularly fascinating. In particular, the activation of prosthetic titanium implant components before their installation in the oral cavity could represent a very advantageous field of application of the argon plasma treatment. Recent studies, in this sense, have suggested that treatment of titanium abutments by argon plasma may enhance peri-implant soft tissue healing at an early stage . I was associated with the preservation of the marginal bone level over time (5,6).

Another promising field of application of the argon plasma technology could be the therapy of peri-implant disease, through the treatment of contaminated prosthetic components and implant surfaces, once a specific hand piece for chair-side use will be available.

However, so far, the effectiveness of plasma technology to decontaminate/sterilize previously infected titanium implant surfaces has not been proven, neither the effect of different surface textures on such decontamination.

The aim of the present study was to assess the effects of the argon plasma treatment on different titanium implant surfaces (turned, sandblasted/acid-etched and titanium plasma sprayed) previously exposed *in vitro* to bacterial contamination.

## Material and Methods

**Figure 1:**
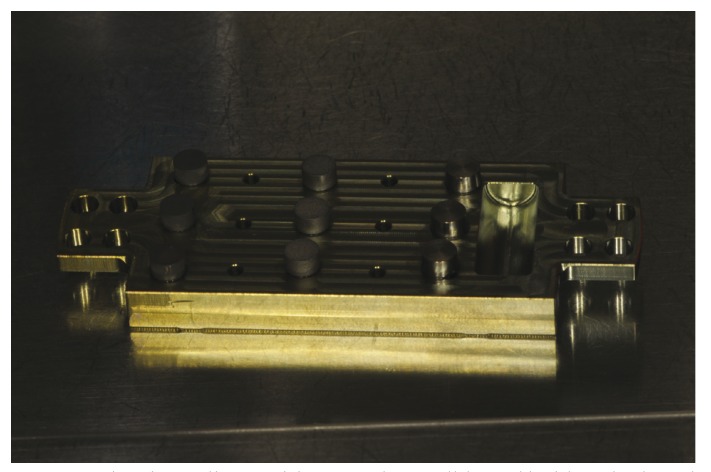
Titanium discs with turned, sandblasted/acid-etched and titanium plasma sprayed surface inserted on a metallic holder and treated in the argon plasma chamber.

Sterile c.p. titanium implant discs (5 mm wide, 3 mm height) with turned (T), sandblasted/acid-etched (SAE) and titanium plasma sprayed (TPS) surface (Sweden & Martina, Padua, Italy) were used in this study. The average roughness (Sa) of these surfaces was 0.8 μm, 1.3 μm and 3.0 μm for T, SAE and TPS discs, respectively. A strain of Aggregatibacter *actinomycetemcomitans* (Aa) ATCC3718 was grown at 37°C under anaerobic conditions for 24 h. 2×106 bacteria cells were inoculated into 48-well flat-bottomed sterile polystyrene micro plates (Costar; Corning, Inc., NY, USA) in which were inserted six discs for each of the three surface types and incubated for 24 h at 37°C. To enhance biofilm formation, discs were previously coated for 1 hour with human saliva. An unstimulated saliva sample was obtained from a healthy male donor, who had not assumed any medication for 3 months prior to the study. The protocol was approved by the Ethics Committee and a specific informed consent was signed by participants. After 24 hours, a half of the contaminated discs (control group) were washed three times with PBS to remove the plank tonic and loosely attached bacteria and transferred to a 15 ml Falcon tube containing 1 ml of PBS. The tubes were sonicated for 1 min at 100% intensity for the disruption of the biofilms. Sonication fluid was diluted and all serial dilutions were then used to evaluate the colony forming units (CFUs). The other half of the contaminated discs (test group) were inserted on a metallic holder and treated in an argon plasma chamber (Plasma R, Sweden & Martina, Padua, Italy) for 12 minutes at room temperature (Fig. 1) prior to be washed, sonicated and analyzed for CFU counting as above mentioned. All assays were performed using triplicate samples of each material in 3 different experiments.

## Results

**Figure 2:**
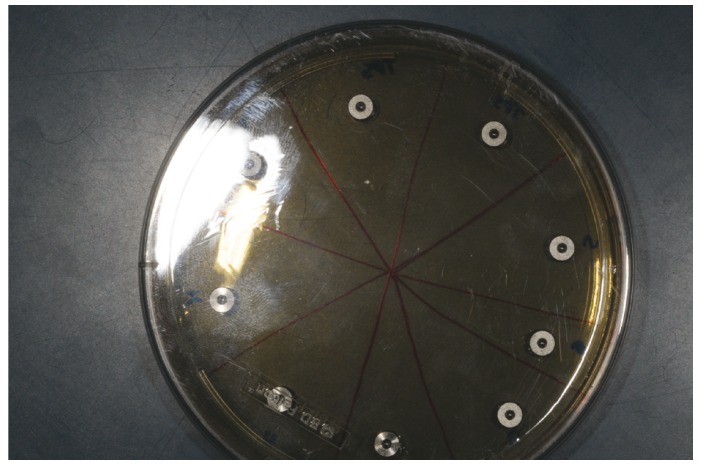
Any trace of bacterial contamination was found on the titanium discs treated in the argon plasma chamber, irrespectively to the implant surface tested.

When the CFU counting was carried out on control discs, a total of 1.50x106±1.4x105, 1.55x106±7.07x104 and 3.15x106±2.12x105 CFU was respectively assessed for T, SAE and TPS discs, without statistically significant differences among the three surfaces. On the contrary, any trace of bacterial contamination was found for the titanium discs treated in the argon plasma chamber prior to be analyzed, irrespectively to the implant surface tested (Fig. 2).

## Discussion

Data from the present *in vitro* study demonstrated that plasma of argon is suitable for removal of any trace of bacterial contamination on previously contaminated titanium discs with different micro-topography.

Aggregatibacter *actinomicetemcomitans* was used as a test microorganism because of its presence in the human oral cavity and in the subgingival biofilm. This microorganism, indeed, is a Gram-negative facultative non-motile rod often found in association with periodontitis, especially in its more aggressive forms (7). At the same time, A. *actinomicetemcomitans* has been shown also to have a role in the peri-implant disease, together with gram-negative anaerobic bacteria commonly associated to chronic periodontitis (Porphyromonas *gingivalis*, Treponema *denticola*, Tannerella *forsythia*, Fusobacterium sp., Prevotella *intermedia*) and microorganisms associated to therapy-resistant (refractory) periodontitis (Staphylococcus *aureus*, Candida *Albicans*) (8).

In the present study, the microbiologic analysis of the titanium disks previously contaminated by Aa for 24h and not treated by the argon plasma chamber (control group), demonstrated bacterial adhesion to all surface types: turned, sandblasted/acid-etched and titanium plasma sprayed. In particular, a trend for slightly higher bacterial adhesion on rougher surfaces was observed, but no statistical significance was detected. This finding was not in accordance with the main part of the literature, in which a direct correlation between bacterial adhesion and surface roughness of titanium implants is generally reported (9). However, it is known how such correlation is based on largely descriptive studies and numerous variables may potentially affect the obtained results and make difficult to compare different studies, including the specific surface treatments applied and the absence of a universally accepted classification system for implant surface features.

According to the data reported, a 12 minute-cycle in the argon plasma chamber allowed to eliminate any trace of bacteria from the three surfaces adopted in the study, irrespectively of their different surface roughness. This is in accordance with Yu *et al*. (10) who demonstrated that the argon plasma is effective in the inactivation/sterilization of surfaces from oral bacteria, and Youngblood & Ong (11), who suggested the use of plasma-glow discharge as an alternative sterilization procedure for medical and dental implants.

Actually, the term “sterilization” refers to any process that eliminates/kills all microorganisms, including bacteria, spores, fungi, viruses and prions. Although few data are available about their effect on prions, which still remain the hardest challenge for any medical sterilization procedure, several studies have shown the ability of cold atmospheric plasmas to promote the decontamination of inanimate objects, thanks to their high anti microbial efficacy and their easy access into narrow and confined spaces (12,13).

The possible mechanism underlying the antibacterial effect of the non-thermal low temperature atmospheric argon plasma has been extensively studied over the past decade (14). It was concluded that effective inactivation of micro-organisms is based on plasma-generated highly reactive agents including UV photons, oxygen species, charged particles as well as electric fields (15).

The main limitation of the present study was represented by the fact that only one bacterial species was used to contaminate disks. Together with the short time of incubation, this did not allow the formation of a well structured biofilm, and further *in vitro* and ex vivo studies are needed to confirm the encouraging, although preliminary, obtained results.

## Conclusions

Within the limit of this study, reported data suggested that the argon plasma technology could be efficiently used to decontaminate/sterilize previously infected titanium implant surfaces. Such encouraging results potentially widen the use of this technology also for the decontamination of infected rough implant surfaces, with the aim to positively affect the response of soft and hard peri-implant tissues. It might open to new possible strategies in the field of peri-implantitis therapy once a specific handpiece for chair-side use would be available.
